# Comparison of 2940 nm Er: YAG laser treatment in the microlaser peel, fractional ablative laser, or combined modes for the treatment of concave acne scars

**DOI:** 10.1097/MD.0000000000026642

**Published:** 2021-07-16

**Authors:** Lei Chen, Yanjin Wang, Lili Jiang, Jing Fang, Jizhen Ren

**Affiliations:** a The Affiliated Hospital of Qingdao University; b The Department of Plastic and Cosmetic Surgery; c Department of Anesthesiology, The Affiliated Hospital of Qingdao University, Qingdao, Shangdong Province, China.

**Keywords:** concave acne scar, Er:YAG laser, fractional ablative, microlaserpeeling

## Abstract

**Objective:**

To compare and analyze the effects of Er:YAG laser treatment in the microlaser peeling, fractional ablative laser, or combined modes for the treatment of concave acne scars.

**Method:**

Ninety patients of concavity acne scar were randomly assigned to three different groups:microlaserpeeling mode group (MM group), fractional ablative mode group (FM group) and combined mode group (CM group). MM group received microlaserpeeling mode with depth of 60 μm and a repetition rate of 20%, FM group received fractional ablative mode with depth of 300 μm and a fractional density of 8%, and CM group received a fractional depth of 200 μm, density of 8%, and a peeling depth of 30 μm, repetition rate of 20%. All patients were evaluated for their treatment effects and side effects 30 days after treatment, including the treatment satisfaction, the ECCA grading scale, pain score and pigmentation level.

**Results:**

According to the effect satisfaction of patients’ self-assessment, the difference among the three groups was statistically significant (*P* < .05), the CM group was better than the other two groups, but there was no significant difference between the FM group and the MM group (*P* > .05). About the ECCA grading scale 30 days after treatment, the statistical result among the three groups was significant (*P* < .05), the CM group is much lower than the FM group which is approximately equal to the MM group. There was statistical difference in pain score among the three groups and every two groups (*P* < .05), the CM group had the highest pain score, while FM group had the lowest. About the pigmentation level, there was statistical difference among the three groups (*P* < .05), FM group had the lightest pigmentation, while the CM group had the heaviest.

**Conclusions:**

Three treatment modes are all effective in treating the concavity acne scar. Among the three modes, CM group is best effective, also accompanied with the most severe side effect; FM group achieves the best balance between treatment effect and side effect. The treatment practices indicate that when the Er:YAG laser with a wavelength of 2940 nm is used to treat concavity acne scars, the right treatment mode should be subject to the severity of the scar.

## 1 Introduction

Acne is a common pilosebaceous chronic inflammatory disease. The triggering factors include endocrine dyscrasia, exuberant sebaceous gland secretion, sebaceous duct obstruction, and bacterial infection. Acne commonly occurs on the face and back of the upper chest,^[1]^ and teenagers are the most affected age group. Several researchers agree that acne scars can be composed of atrophic and/or hypertrophic scars. Clinically, atrophic scars are the most prominent and are divided into three types depending on their shape: ice pick, boxcar, and rolling.^[2–3]^ Currently, there are multiple treatments for atrophic scars. Among them, treatment with Er:YAG lasers at a 2940 nm wavelength shows a relatively satisfactory effect. This study aimed to assess the clinical treatment effects of the Er:YAG lasers.

## 2 Data and methods

### 2.1 Data

#### 2.1.1 Ethics and consent

Ethical approval for this randomized, controlled study was provided by the Ethical Committee of the Affiliated Hospital of Qingdao University, Shandong, China. Informed consent was obtained from all patients enrolled in the study.

Ninety patients diagnosed with concave acne scars between February 2016 and October 2018 were selected. These patients volunteered to participate in the study and signed the informed consent. The study group included 29 men and 61 women age between 18–35 years and an average age of 27.5 years. The follow-up time for these patients ranged from 2 to 12 years, with an average of 5 years. The mixed skin lesions in all patients were located on the face, with areas ranging from 20 cm^2^ to 55 cm^2^. The skin lesions were present in all patients for >6 months. According to the Fitzpatrick scale, these patients had skin types of III and IV. All patients were randomly divided into three groups, each group comprising 30 patients, and were treated with FM, MM, and CM once-time respectively.

#### 2.1.2 Inclusion criteria

1. presence of concave acne scars for >6 months,
2. comprehension of the purpose and course of treatment, and
3. consent for documentation with relevant photographs and additional information and a signed informed consent form.

#### 2.1.3 Exclusion criteria

1. patients with unreasonable expectations in terms of treatment effect;
2. patients using corticosteroid hormone drugs, receiving anticoagulant therapy, or having photosensitivity reactions when such drugs or foods were administered or directly exposed to strong sunshine after using such drugs or foods possibly leading to photosensitivity reaction;
3. patients using tretinoin drugs through external application;
4. patients with metabolic diseases;
5. patients who were pregnant, lactating, or had hypertrophic scarring;
6. patients with epidermal cancer, severe hepatic failure, renal failure, and other catastrophic diseases;
7. patients who had undergone facial esthetic treatment that might have affected the treatment in the six months before laser treatment, including blepharoplasty, botulinum toxin injection, dermabrasion, exfoliation to promote blood circulation, laser operation, or rhytidectomy;
8. patients with active herpes simplex, dermatitis, and other active dermatosis;
9. patients with active psoriasis, vitiligo, and severe eczema, easily leading to a isomorphic response;
10. patients implanted with a metal apparatus on his/her skin or bone (steel plate or nail);
11. patients with abnormal pigmentation levels before treatment;
12. patients with diseases that could hamper wound healing or cause diminished skin healing, e.g., active infection, immunosuppression, blood coagulation dysfunction, and other diseases in the blood or peripheral blood vessels;
13. patients with a cardiac pacemaker or with a history of similar implants;
14. patients suffering from neuropathy or mental illness.^[4]^

### 2.2 Treatment equipment

The Er:YAG laser (Sciton, USA) used for concave acne scars has two different modes, microlaser peeling mode (MM) and fractional ablative mode (FM).

### 2.3 Treatment method

In our study, 90 patients were selected to receive treatment under one of three different modes (MM, FM, and their combined mode [CM]). The treatment effect was compared and analyzed 30 days after treatment.

An informed consent form was signed before treatment. After removing all cosmetics, the face was washed and the skin was disinfected. After a single treatment, a designated staff member used the same camera to take a photograph of the patient with the same optical source from the same angle and filed the photographs for evaluation of the treatment effects in the future. Lidocaine cream (5%) was applied at the partial treatment area as an anesthetic. A piece of plastic wrap was used to seal the compound lidocaine cream for half an hour. Half an hour later, fresh water was used to remove the compound lidocaine cream and then the patient's face was wiped dry. Bromogeramine was applied for sterilization. Patients lay on their back and wore safety goggles. Treatment parameters were as follows: in the MM group, depth 60 μmand a repetition rate of 20%; In the FM group, a depth 300 μm and fractional density of 8%; in the CM group, fractional depth 200 μm, fractional density 8%, peeling depth 30 μm, and repetition rate 20%. After adjusting the treatment parameters to an appropriate level, the doctor wore protective goggles and initiated treatment. Patients were instructed to avoid face contact with water for three days from treatment commencement and to avoid exposure to strong sunshine. If going outdoors was necessary, patients were advised to lay on their backs, wear safety goggles, a cap, and sunglass; and to apply other sun protection products. For 7 days from treatment commencement, moist exposed burn ointment and recombinant bovine basic fibroblast growth factor gel were applied onto the facial skin of the patient twice daily. The patient was instructed to avoid using isotretinoin, vitamin A, and alpha hydroxy acid for 1–2 weeks after treatment. Immediately after treatment, on the first and third day after operation, each patient completed a McGill pain questionnaire for evaluation of pain. Thirty days later, the pigmentation level of the patients was evaluated.

### 2.4 Methods to evaluate treatment effect

#### 2.4.1 ECCA for evaluation of treatment effect before and after treatment

A camera (Canon 6D; Canon Tokyo, Japan) was used to click photographs from the front,45° left, and right positions for evaluation before treatment and at follow-up. All photographs were taken with the same location, angle, posture, shooting location, light source, and equipment and by the same photographer. Two specially designated doctors used Dreno's ECCA grading scale to score the patients’ photographs within 30 days before and after the treatment. The scoring criteria were based on the atrophic scar characteristics and density as follows:

1. Value a: with a diameter <2 mm, a weight score of 15; Boxcar shape atrophic scar, also referred to as U-shape or boxcar-shape scar: with a diameter of 2–4 mm, sharp edges, a weight score of 20; Rolling shape atrophic scar, also referred to as M-shape or rolling scar: with a diameter >4 mm, mostly irregular edges, a weight score of 25;
2. Value b: score based on the density of the atrophic scar: score 0 with no scar existent (scar number≦5), score 2 with limited number of scars (5<scar number≦20), score 3 with a large number of scars (<20);
3. Final score of scars: value a × value b.

The score obtained was based on the property, density of the scars and quantitatively reflected the severity level of the atrophic scar to some extent.

#### 2.4.2 Self-evaluation of treatment satisfaction by the patient

The patient was asked to evaluate the treatment effect 30 days after treatment completion. The satisfaction had four levels: excellent, good, ordinary, and poor. Satisfaction degree = (patients marking excellent + patients marking good)/total number of patients × 100%.

#### 2.4.3 Record of clinical side effects

The patient was asked to evaluate the degree of pain using the visual analogue scale (VAS) during treatment, on the first day post treatment, and on the third day post treatment (0 means minimum value and indicates no pain existent; 1–3 means mild pain and indicates that sleep was not disturbed;4–6 means moderate pain and indicates disturbance of sleep;7–10 means severe pain and indicates that the patient was unable to sleep because of pain; 10 means maximum value and indicates sharp pain). The doctor evaluated the pigmentation levels immediately after treatment completion and 30 days after completion. The evaluation score ranged from 0 to 3, with the highest being a score of 3, indicating severe pigmentation.

### 2.5 Statistical analysis

The statistical software SPSS 22.0 was used for analysis. The paired *t*-test was used to indicate the change in the ECCA score before and after treatment for each group. Analysis of variance was used to compare the ECCA scores between any two groups after treatment. The rank-sum test was applied to evaluate the satisfaction with treatment on the basis of patient self-evaluation. Analysis of variance was used for comparison of statistical data on side effects (pain and pigmentation) between any two groups. *P* values <.05 was considered statistically significant.

## 3 Results

### 3.1 ECCA score used for evaluation of treatment effects before and after treatment (Fig. 1)

The ECCA score of all patients before treatment were similar, with no statistically significant difference (*P* > .05). The ECCA scores of patients after treatment in each group were significantly reduced compared to those before treatment (*P* < .01). The ECCA score of the CM group was significantly lower than that of the MM and FM groups (*P* < .01). There was no difference between the ECCA score of the MM and FM groups (*P* > .05).

**Figure 1:**
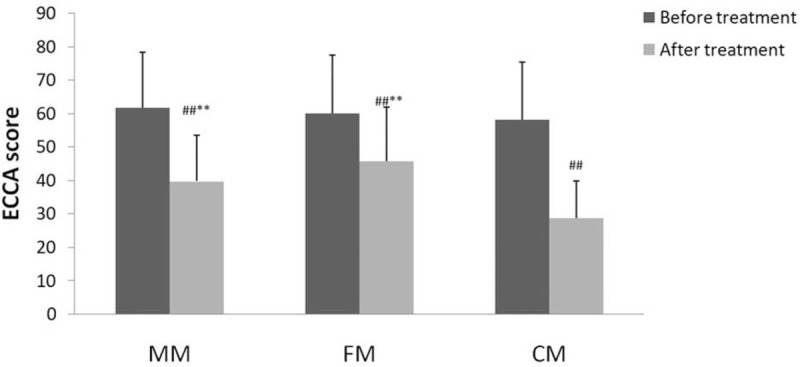
ECCA scores of treatment effect on atrophic scars. Data are presented as mean±SEM, n = 30. ^∗∗^*P* < .01 vs the CM group; ^##^*P* < .01 After treatment vs before treatment in each group. CM = combined mode.

### 3.2 Self-evaluation of treatment satisfaction by patients (Table 1)

Ninety patients underwent the treatment, completed , and accepted the evaluation. The FM group had a satisfaction rate of 53.3%; the MM group, 46.7%; and the CM group, 83.3%. The satisfaction rate of the CM group was statistically higher than that of the other two groups (*P* < .01); however, there was no difference between the MM and FM groups (*P* > .05).

**Table 1:** Self-evaluation of treatment satisfaction by patient. Treatment satisfaction of patient by self-evaluation.

Group (n = 30)	Excellent	Good	Ordinary	Poor	Satisfaction degree (%)
MM	5	9	16	0	46.67^∗^
FM	2	14	14	0	53.33^∗^
CM	14	11	5	0	83.33

Data are presented as number of cases and satisfaction rate.

∗ *P* < .01 vs the CM group. CM = combined mode.

### 3.3 Record of clinical side effects

**Figure 2:**
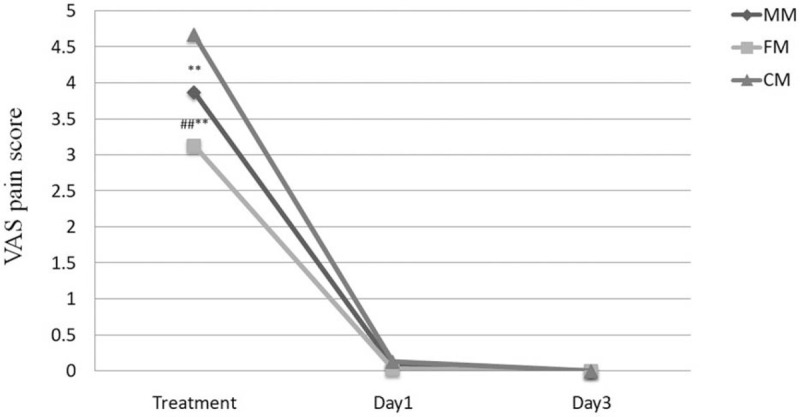
VAS scores of patients during treatment, on the first day after treatment, and on the third day after treatment. Data are presented as mean±SEM, n = 30. ^∗∗^*P* < .01 vs the CM group; ^##^*P* < .01 MM group vs FM group. CM = combined mode, FM = fractional ablative mode, MM = microlaser peeling mode, VAS = visual analogue scale.

The side effects of 90 patients were evaluated. During the course of treatment, VAS scores were significantly higher in all three patients’ group. Furthermore, the VAS score of the CM group was significantly higher than that of the MM and FM groups (*P* < .01), whereas the VAS score of the MM group was significantly higher than that of the FM group (*P* < .01). On the first day after treatment, the VAS scores were significantly lower in all three groups, and the patients did not have significant pain. On the third day after treatment, all patients with VAS scores zero did not experience pain (Fig. 2).

**Figure 3:**
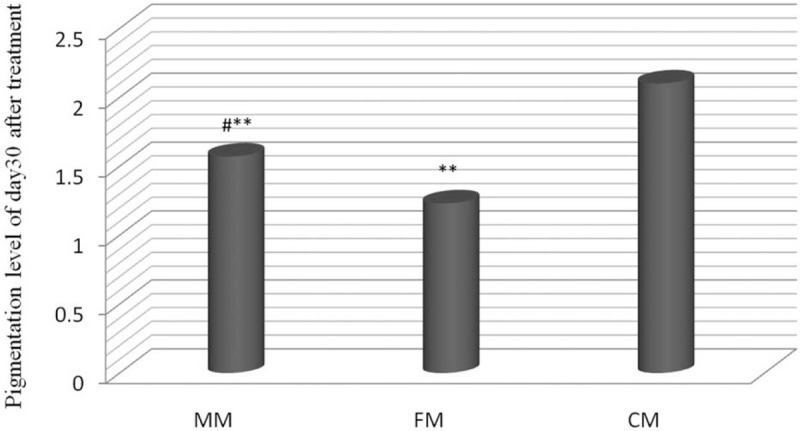
Pigmentation score on day 30 after treatment. Data are presented as mean±SEM, n = 30. ^∗∗^*P* < .01 vs the CM group; ^#^*P* < .05MM group vs FM group. CM = combined mode, FM = fractional ablative mode, MM = microlaser peeling mode.

No patient developed abnormal pigmentation, and the scores assessed by the doctor were zero during treatment. On day 30 after treatment, the pigmentation level of the MM group was significantly higher than that of the CM group (*P* < .05), whereas the pigmentation level of the CM group was statistically higher than that of the MM and FM groups (*P* < .01) (Fig. 3).

## 4 Discussion

A scar is the natural result of repair after trauma to the body. It is formed because of the falsification of the collagen structure in the corium layer and abnormal hyperplasia of the myofibroblast during repair after trauma. Acne is a clinically common type of chronic skin inflammation. The prognosis indicates that patients with severe acne usually have concave acne scars. Acne scars not only disfigure patients but also seriously impact their self-confidence, job, life, and social interactions. These scars require early treatment.^[5]^

Currently, there are various approaches to improve acne scars, including skin dermabrasion, chemical peeling, cicatrectomy, and laser therapy. An Er:YAG laser with a wavelength of 2940 nm is a relatively popular clinical tool used to eliminate acne scars. It functions by promoting regeneration and rearrangement of skin collagen.^[6]^ The Er:YAG laser has a relatively high water absorptivity,^[7]^ 10–20 times higher than that of a carbon dioxide laser. Therefore, it is more accurate, with less thermal injury to the surrounding tissues and a shorter repair time.^[8]^ Moreover, its’ heat effect on the dermis may ease local inflammation and reduce the formation of post-acne scars.^[9]^ An Er:YAG laser with a wavelength of 2940 nm has two working modes, MM and FM. The former indicates that the laser to be fully absorbed by the water in the skin so that the skin can be removed instantly from outside to inside. Thus, it accurately removes the epidermis and dermis with a maximum depth of 40 μm. It assures safe formation of new skin and regeneration of collagen. The FM utilizes focal photothermy to produce rays of micro-light beams that act on the skin. After the water in the skin tissue absorbs laser energy, more than one column-shaped thermal injury zone will be formed, called the micro-thermal zone. Such an array of thermal stimulation can uniformly initiate skin repair for reshaping and rebuilding full-thickness skin, including the epidermis and dermis, for treatment.^[10]^

**Figure 4:**
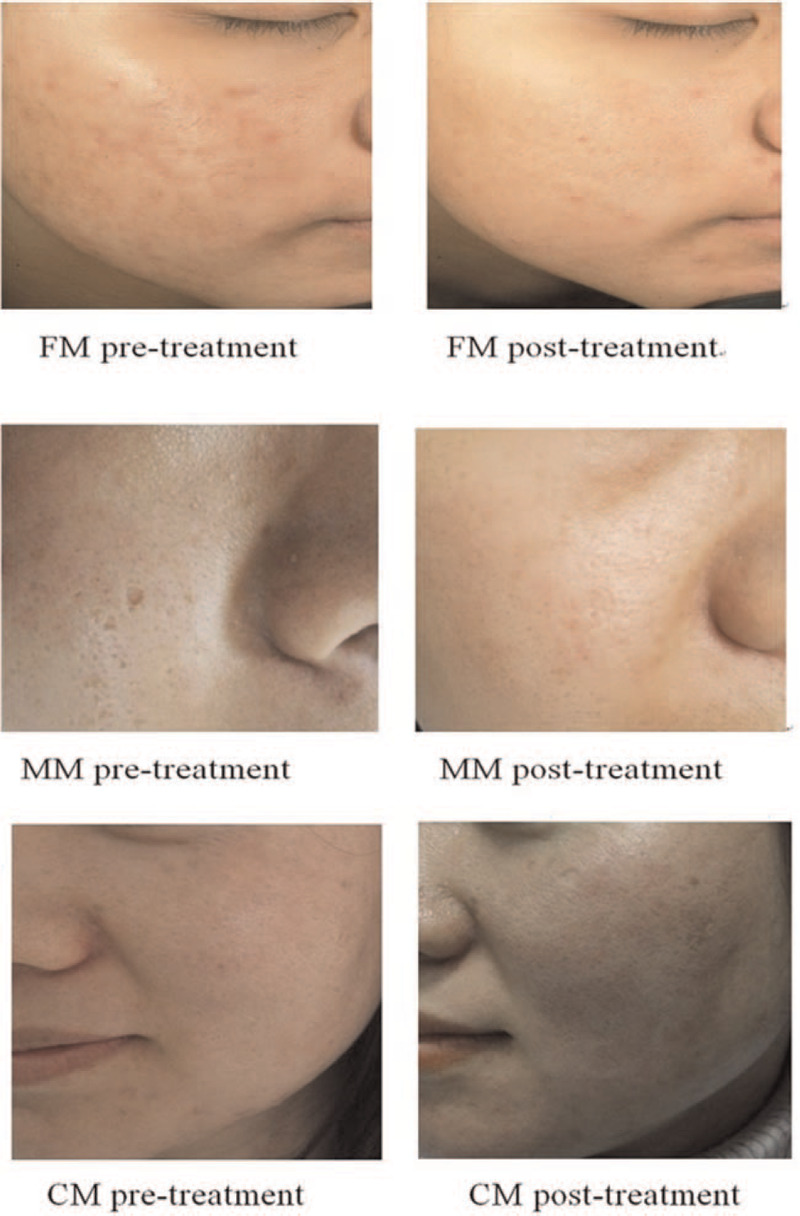
The therapeutic effect of three treatment modes.

The ECCA scores in all three groups were reduced to some extent after treatment, the scores being statistically significant on comparison (*P* < .05), which proves that all three treatment modes were clinically effective (Fig. 4). Compared with CM group, FM group had a higher mean ECCA score, and the difference was statistically significant. This indicates that the former allowed better treatment. FM group had a higher mean ECCA score than MM group; however, there was no statistical difference between the two groups, indicating no effective difference between them.

As for the pain score, FM group had a lower mean pain score than MM group, and the difference was statistically significant, which indicated that the patient treated with FM had a lower degree of pain. MM group had lower mean pain score than CM group, and the difference was statistically significant, which indicated that the patient treated with MM had a lower degree of pain. On the first day after operation, 7.8% of patients had a pain evaluation score of 1, whereas the rest had a score of 0. On the third day after the procedure, all patients had a pain evaluation score of 0. This proved that the pain of most patients only appeared on the first day after operation, irrespective of the treatment mode applied.

With regard to pigmentation, FM group had a lower mean pigmentation score than MM group, and the difference was statistically significant, which indicated that FM led to lower pigmentation than MM. MM group had a lower mean post-treatment 30-day pigmentation level than CM group, which indicated that MM resulted in a lower pigmentation level than CM.

In conclusion, this study proves that the three treatment modes of Er:YAG laser treatment with a wavelength of 2940 nm are all effective for the treatment of concave acne scars. Among the treatment modes, CM showed the best treatment effect. However, its side effects and complications are also the most severe, including pain and post-operative pigmentation. FM is inferior to CM but superior to MM. In contrast, FM has weakest side effects, including pain and post-operative pigmentation. It could be concluded that FM features the best balance between satisfactory treatment and side effects. Meanwhile, according to the different scar areas of patients, it is feasible to combine multiple treatment methods. For example, CM is relatively effective if the patient has a severe scarring. In contrast, FM could be applied to in patients with relatively mild scaring. Considering the relatively severe pain reaction in CM, we suggest administering supraorbital and infraorbital nerve block anesthesia to reduce pain during treatment. Subsequently, we will conduct further research on alleviating pain during treatment, promoting healing of the wound surface, and achieving individual and acceptable treatment.

In this study, we used the random number table method to randomly group the patients and conduct data statistics and data analysis in a scientific and rigorous manner. We tried to minimize the influence of bias and confounding factors on the results of the study. But this study also has limitations. The sample size of this study is small, and the blind method is not used in the course of the study. These factors may affect the results of the study. Therefore, our conclusions still need to be further verified by scientific and rigorous clinical trials.

## Author contributions

**Conceptualization:** Yanjin Wang, Jing Fang, Jizhen Ren.

**Data curation:** Lili Jiang.

**Resources:** Yanjin Wang, Jizhen Ren.

**Software:** Lili Jiang.

**Writing – original draft:** Lei Chen.

**Writing – review & editing:** Lei Chen, Lili Jiang.

## References

[1] Bei H Luo W Liu P. Aesthetic analysis of applying fractional photothermolysis to treat concavity acne scar. Clin Med Engin 2015;22:1589–90.

[2] Goodman GJ. Postacne scarring:a review of its pathophysiology and treatment. Dermatol Surg 2000;26:857–71. 10971560 10.1046/j.1524-4725.2000.99232.x

[3] Jacob CI Dover JS Kaminer MS. Acne scarring:a classification system and review of treatment option. J Am Acad Dermatol 2001;45:109–17. 11423843 10.1067/mjd.2001.113451

[4] Zheng X. Effect of using fractional photothermolysis to treat acne scar respectively with a laser wavelength of 2940 nm and 1550 nm and safety evaluation. Guangzhou: Southern Medical University; 2015.

[5] Manuskiatti W Iamphonrat T Wanitphakdeedecha R. Comparison of fractional erbium-doped yttrium aluminum garnet and carbon dioxide lasers in resurfacing of atrophic acne scars in Asians. Dermatol Surg 2013;39:111–20. 23205717 10.1111/dsu.12030

[6] Dierickx CC Khatri KA Tannous ZS. Micro-fraction ablative facial skin resurfacing with the two novel erbium laser systems. Lasers Surg Med 2008;40:113–23. 18306165 10.1002/lsm.20601

[7] Magnani LR Schweiger ES. Fractional CO2 lasers for the treatment of atrophic acne scars: a review of the literature. J Cosmet Laser Ther 2014;16:48–56. 24131097 10.3109/14764172.2013.854639

[8] Sardana K Garg VK Arora P. Histological validity and clinical evidence for use of fractional lasers for acne scars. J Cutan Aesthet Surg 2012;5:75–90. 23060702 10.4103/0974-2077.99431 PMC3461801

[9] Cheng X Liu S Zhou B. Current situation of researches about using CO2 microlaserpeeling fractional photothermolysis and 1550 nm non-microlaserpeeling fractional photothermolysis to treat acne scars. Chinese J Aesth Plastic Surg 2017;28:187–9.

[10] Liang H. Application of Er:YAG laser with a wavelength of 2940 nm at general face cosmetology strategy. Chinese J Laser Med Surg 2015;23:265.

